# Three New Cembranoids from the Taiwanese Soft Coral *Sarcophyton ehrenbergi*

**DOI:** 10.3390/md10071433

**Published:** 2012-06-27

**Authors:** Shang-Kwei Wang, Mu-Keng Hsieh, Chang-Yih Duh

**Affiliations:** 1 Asia-Pacific Ocean Research Center, National Sun Yat-Sen University, Kaohsiung 804, Taiwan; Email: skwang@cc.kmu.edu.tw; 2 Department of Microbiology, Kaohsiung Medical University, Kaohsiung 807, Taiwan; 3 Department of Marine Biotechnology and Resources, National Sun Yat-Sen University, Kaohsiung 804, Taiwan; Email: m995020015@student.nsysu.edu.tw

**Keywords:** *Sarcophyton ehrenbergi*, cembranoids, cytotoxicity, anti-HCMV

## Abstract

In order to search for new bioactive substances from marine organisms, we have investigated the acetone extracts of the soft coral *Sarcophyton ehrenbergi* collected at San-Hsian-Tai, Taitong County, Taiwan. Chromatographic fractionation of the extracts of the octocoral *S. ehrenbergi* led to the isolation of three new cembranoids, (+)-12-ethoxycarbonyl-11*Z*-sarcophine (**1**), ehrenbergol A and B (**2** and **3**). The structures of these isolated metabolites were elucidated through extensive spectroscopic analyses. Moreover, metabolites **1**–**3** were evaluated *in vitro* for their cytotoxicity towards selected cancer cell lines and antiviral activity against human cytomegalovirus (HCMV).

## 1. Introduction

Marine organisms, which have developed unique metabolic and physiological capabilities to ensure survival in extreme ocean habitats, offer the potential to produce new bioactive constituents that would not be observed from terrestrial organisms [1]. Soft corals belonging to the genus *Sarcophyton* (Alcyoniidae) have been well recognized as a rich source of terpenoids [1]. These constituents, mainly macrocyclic cembrane-type diterpenoids and their derivatives, represent important chemical defense substances for the animals against their natural predators [2]. Cembranoids have been previously reported to exhibit a range of biological activities including antitumor [3,4,5,6,7,8,9], ichthyotoxic [10], anti-inflammatory [11], neuroprotective [12], antibacterial [13], antiangiogenic [14], antimetastatic [14], anti-osteoporotic [15], and cytotoxic [16,17,18] properties. Among them, sarcophine (**4**) was reported to have antimetastatic activity [14].

**Figure 1:**
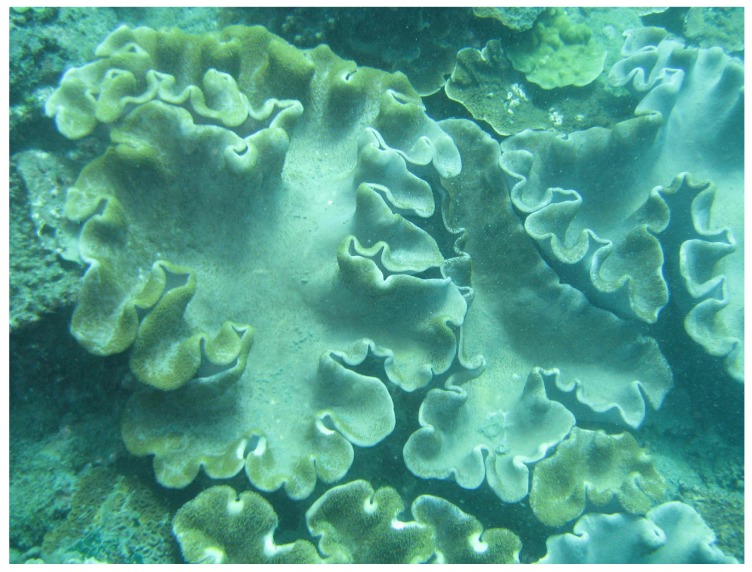
Soft coral *Sarcophyton ehrenbergi*.

**Figure 2:**
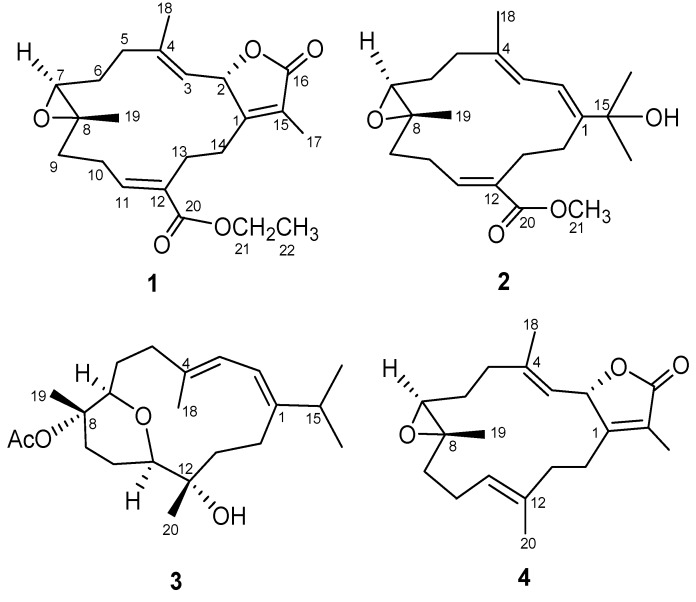
Structures of compounds **1**–**4**.

Twelve cembranoids were previously reported from the soft coral *Sarcophyton ehrenbergi* [1,19]. The samples for our previous studies on the secondary metabolites of the soft coral *S. ehrenbergi* were all collected at Dongsha Atoll [19,20]. Chemical investigation of the Taiwanese soft coral *S. ehrenbergi* (Figure 1) collected at San-Hsian-Tai (Taitong County) has afforded three new cembranoids, designated as (+)-12-ethoxycarbonyl-11*Z*-sarcophine (**1**), ehrenbergol A and B (**2** and **3**) (Figure 2). Herein, we describe the purification, structure elucidation, cytotoxicity and antiviral evaluation of these metabolites in detail.

## 2. Results and Discussion

The HRESIMS of **1** exhibited a pseudomolecular ion peak at *m/z* 397.1993 [M + Na]^+^, consistent with the molecular formula of C_22_H_30_O_5_, requiring eight degrees of unsaturation. IR absorption at 1754 cm^−1^ and NMR signals (Table 1) at *δ*_C_ 174.4 (qC, C-16), 160.7 (qC, C-1), 124.2 (qC, C-15), 78.2 (CH, C-2), and 8.6 (CH_3_, C-17); *δ*_H_ 5.57 (1H, dd, *J* = 10.0, 2.0 Hz, H-2) and 1.90 (3H, s, H_3_-17) were indicative of an α,β-unsaturated γ-lactone functionality by comparison with those of similar metabolites, such as the corresponding data of sarcophine (**4**) [6]. The IR absorption bands at 1705 cm^−1^ and NMR signals at *δ*_H_ 6.80 (1H, dd *J* = 10.4, 4.8 Hz, H-11), 4.23 (2H, m, H_2_-21), 1.32 (1H, t, *J* = 7.2 Hz, H-22); *δ*_C_ 166.9 (qC, C-20), 131.1 (qC, C-12), and 142.0 (CH, C-11) indicated the presence of α,β-unsaturated ethyl ester [20]. In addition, a trisubstituted epoxide was present in **1** from its ^1^H NMR signals at *δ*_H_ 2.56 (1H, br d, *J* = 6.4 Hz, H-7) and ^13^C NMR signals at *δ*_C_ 61.3 (qC, C-8) and 62.4 (CH, C-7). Moreover, the ^13^C NMR signals at *δ*_C_ 121.1 (CH, C-3), and 144.5 (qC, C-4) were assigned a trisubstituted double bond. The above functionalities account for seven of the eight degrees of unsaturation, suggesting a tricyclic structure in **1**.

**Table 1:** NMR data for compound **1**.

Position	*δ*_H_ *^a^* (*J* in Hz)	*δ*_C_ *^b^*, Type	HMBC	COSY	NOESY
1		160.7, C			
2	5.57, dd (10, 2.0)	78.2, CH		3, 17	18
3	5.08, d (10.4)	121.1, CH	5, 18	2, 18	5a
4		144.5, C			
5a	2.39, m	37.7, CH2	3, 4, 6, 18	5b, 6a	3, 5b, 7
5b	2.41, m		3, 4, 6, 18	5a, 6a	5a,18
6a	1.92, m	22.9, CH_2_		6b, 5a, 5b	6b
6b	1.72, m			6a, 7	6a,18, 19
7	2.56, br d (6.4)	62.4, CH	6	6b	9a, 10a
8		61.3, C			
9a	0.97, m	38.2, CH_2_	10	9b	9b, 11
9b	2.22, m		11	9a, 10a, 10b	7, 9a, 19
10a	2.11, m	25.9, CH_2_	12	9a, 10b	7, 10b
10b	2.19, m			9a, 10a, 11	10a
11	6.80, dd (10.4, 4.8)	142.0, CH	10, 13, 20	10a	9b
12		131.1, C			
13	2.36, m	25.2, CH_2_	12, 20	14b	
14a	2.50, m	27.0, CH_2_	1, 2, 15	14b	14b
14b	2.11, m			13a, 14a	14a
15		124.2, C			
16		174.4, C			
17	1.90, s	8.6, CH_3_	1, 15, 16	2	
18	1.87, s	15.3, CH_3_	3, 4, 5	3	2, 5b
19	1.30, s	16.8, CH_3_	7, 8, 9		6b, 9b
20		166.9. C			
21	4.23, m	60.8, CH_2_	20	22	22
22	1.33, t (7.2)	14.3, CH_3_	21	21	21

*^a^* Spectra were measured in CDCl_3_ (400 MHz); *^b^* Spectra were measured in CDCl_3_ (100 MHz).

**Figure 3:**
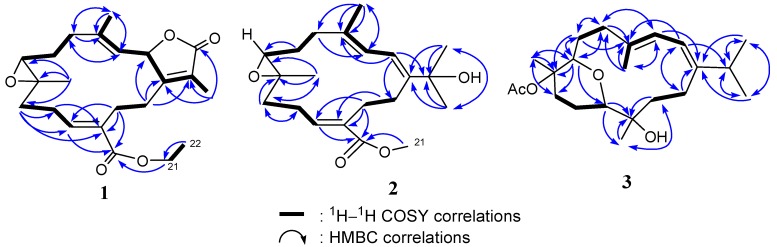
COSY and HMBC correlations of compounds **1**–**3**.

By interpretation of ^1^H-^1^H COSY correlations, it was possible to establish three partial structures of consecutive proton systems extending from H-2 to H-3, from H_2_-5 to H-7 through H_2_-6, from H_2_-9 to H-11 through H_2_-10, and from H_2_-13 to H_2_-14. Subsequently, the connectivities of these partial structures were further established by the HMBC correlations (Figure 3). HMBC correlations observed from H_3_-19 to C-7, C-8, and C-9 indicated the position of the epoxide at C-7 and C-8. Moreover, the HMBC correlations from H-11 to C-9, C-10, C-12, C-13, and C-20 and from H_2_-21 to C-20 as well as COSY correlation between H_2_-21 and H_3_-22, led the assignment of the ethoxycarbonyl at C-12. The locations of the double bond at C-3/C-4 was clarified by analysis of the HMBC correlations from Me-18 to C-3, C-4, and C-5. The molecular framework of **1** was further established by other HMBC correlations between H_2_-14 to C-1, C-2, C-15 and H_3_-17 to C-1, C-15, C-16.

**Figure 4:**
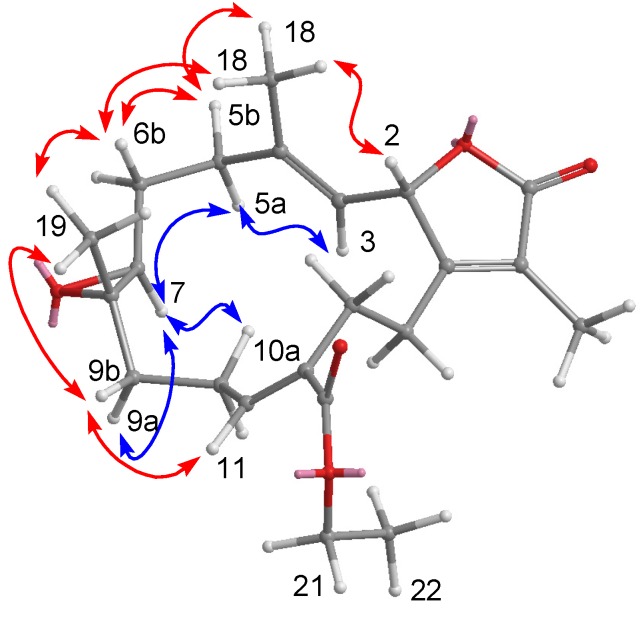
NOESY correlations of compound **1**.

The relative configuration of **1** assigned by a NOESY spectrum was compatible with that suggested by computer modeling, in which the close contacts of atoms calculated in space were consistent with the NOESY correlations (Figure 4). The presence of a NOESY cross peak between the vinylic H-11 and H_2_-9 (*δ*_H_ 2.22) suggested the *E* geometry for the C-11/C-12 double bond, which was also identified by the chemical shift of H-11 at *δ*_H_ 6.80 [19,21]. The geometry of the trisubstituted olefin at C-3/C-4 was assigned as *E* based on the higher field chemical shift of the olefinic methyl signal for C-18 (*δ*_C_ 15.3). Furthermore, the crucial NOE correlations between H-2/Me-18, Me-18/H-6b (*δ*_H_ 1.72), Me-19/H-6b, Me-19/H-9b (*δ*_H_ 2.22), H-7/H-6a (*δ*_H_ 1.92), and H-7/H-9b, H-7/H-5a (*δ*_H_ 2.39), and H-3/H-5a demonstrated the 2S*, 7S*, and 8S* configurations as depicted in Figure 4. A careful analysis of all the NMR spectroscopic data (COSY, HSQC, HMBC, and NOESY) confirmed that **1** is actually the 12-ethoxycarbonyl derivative of (+)-11*Z*-sarcophine [19,22]. All of the NMR spectroscopic data of **1** were consistent with the structure shown as (+)-12-ethoxycarbonyl-11*Z*-sarcophine.

Ehrenbergol A (**2**) was assigned a molecular formula of C_21_H_32_O_4_, according to its HRESIMS and NMR spectroscopic data (Table 2). The IR absorptions of **2** at 1715 cm^−1^ revealed the presence of an α,β-unsaturated methyl ester functionality, which was confirmed by its NMR spectroscopic data [*δ*_H_ 6.89 (1H, dd, *J* = 10.4, 6.8 Hz, H-11) and 3.42 (3H, s, H_3_-21); *δ*_C_ 167.6 (qC, C-20), 133.9 (qC, C-12), 141.1 (CH, C-11), and 51.2 (CH_3_, COOMe)]. The NMR spectroscopic data also indicated that **2** possesses a trisubstituted epoxide [*δ*_H_ 2.67 (1H, dd, *J* = 10.8, 2.8 Hz, H-7); *δ*_C_ 60.6 (qC, C-8) and 62.1 (CH, C-7)], and two trisubstituted olefins [*δ*_H_ 6.53 (1H, d, *J* = 11.0 Hz, H-2) and 6.08 (1H, d, *J* = 11.0 Hz, H-3); *δ*_C_ 147.1 (qC, C-1), 118.4 (CH, C-2), 123.8 (CH, C-3), and 135.8 (qC, C-4)]. The above functionalities account for five of the six degrees of unsaturation, suggesting that **2** must consist of a 14-membered ring diterpenoid skeleton.

**Table 2:** NMR data for compound **2**.

Position	*δ*_H_ *^a^* (*J* in Hz)	*δ*_C_ *^b^*, Type	HMBC	COSY	NOESY
1		147.1, C			
2	6.53, d (11.0)	118.4, CH	4, 14, 15	3	16, 17, 18
3	6.08, d (11.0)	123.8, CH	5, 18	2, 18	5a, 7, 13a
4		135.8, C			
5a	2.12, m	37.8, CH_2_	3	5b, 18	3, 5b
5b	1.97, m		3	5a, 6b, 18	5a, 6b, 18
6a	1.94, m	25.5, CH_2_	7	6b, 7	6b
6b	1.17, m			5b, 6a, 7	5b, 6a,19
7	2.67, dd (10.8, 2.8)	62.1, CH		6a, 6b	3, 9a
8		60.6, C			
9a	0.85, td (12.4, 4.0)	39.7, CH_2_	8, 19	9b, 10a, 10b	9b, 7
9b	1.95, m		8, 10, 19	9a, 10a, 10b	9a, 19
10a	2.16, m	26.7, CH_2_		9, 10b, 11	10b
10b	1.75,m		9, 11, 12	9, 10a, 11	10a, 19
11	6.89, dd (10.4, 6.8)	141.1, CH	20	10a, 10b	9
12		133.9, C			
13a	2.26,m	28.4, CH_2_			3, 13b, 14b
13b	2.38, m		11, 12, 20		13a
14a	2.52, m	28.6, CH_2_	12		14b, 17
14b	2.24, m		12		14a
15		73.6, C			
16	1.33, s	29.5, CH_3_	1, 15, 17		2
17	1.44, s	29.3, CH_3_	1, 16, 17		2
18	1.58, s	15.6, CH_3_	3, 4, 5	3, 5a, 5b	2, 5b
19	1.04, s	15.6, CH_3_	7, 8, 9		6b, 9b, 10b
20		167.6, C			
21	3.42, s	51.2, CH_3_	20		

*^a^* Spectra were measured in CDCl_3_ (400 MHz); *^b^* Spectra were measured in CDCl_3_ (100 MHz).

The structure of **2** was established through COSY and HMBC experiments (Figure 3). Crucial HMBC correlations from Me-18 to C-3, C-4, and C-5, from Me-19 to C-7, C-8, and C-9, from H-11 to C-10, C-12, C-13, and C-20, and from Me-16/Me-17 to C-15 and C-1 confirmed the connectivity among these partial structures (Figure 3). The position of the methoxycarbonyl at C-12 was established by the HMBC correlations from H-11 and H-13 to C-20 and from H_3_-21 to C-20. A COSY experiment established a correlation between the two vinylic protons at *δ*_H_ 6.53 (H-2) and 6.08 (H-3). These results allowed the assignment of the planar structure of **2** as shown.

**Figure 5:**
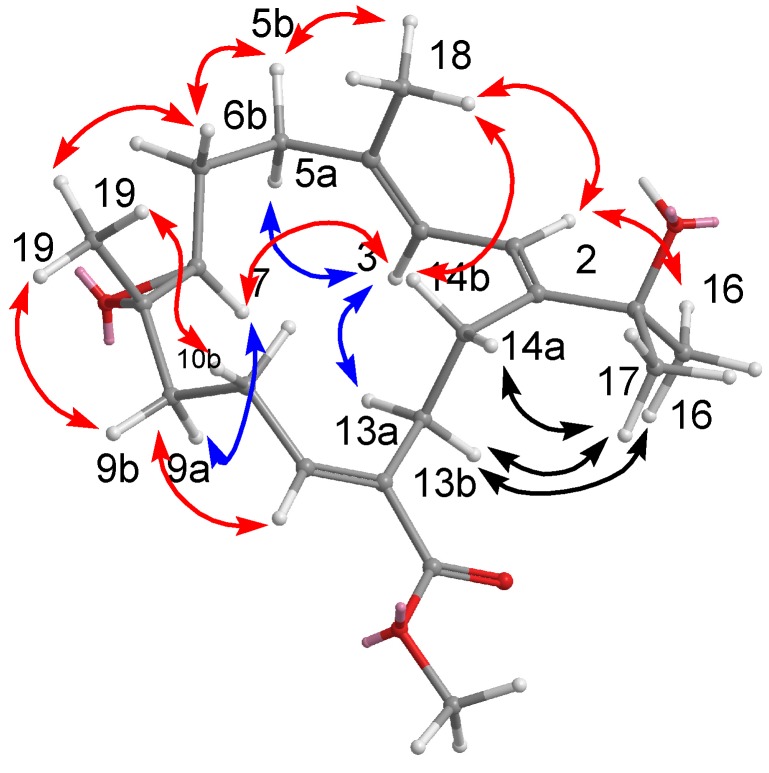
NOESY correlations of compound **2**.

The configurations of all double bonds were determined from a NOESY experiment on **2**. The crucial NOE correlations (Figure 5) between H-2 (*δ*_H_ 6.53)/Me-16 (*δ*_H_ 1.33), H-2/Me-18 (*δ*_H_ 1.44), and H-3 (*δ*_H_ 6.08)/H-5a (*δ*_H_ 2.12) indicated that the geometries of the conjugated diene at C-1/C-2 and C-3/C-4 were both *E*. The large coupling constant (*J*_2,3_ = 11.0 Hz) further suggested the *s*-*trans* geometry of the conjugated double bonds [20,21]. The presence of a NOESY cross peak between the vinylic H-11 and H_2_-9 made it possible to identify the configuration of the olefin at C-11/C-12 as the *E* geometry, which was also confirmed by the chemical shift of H-11 at *δ*_H_ 6.89 [20]. Moreover, the crucial NOESY correlations between Me-19/H-6b (*δ*_H_ 1.16), Me-19/H-9b (*δ*_H_ 1.94), H-7/H-6a (*δ*_H_ 1.97), H-7/H-9b (*δ*_H_ 1.95), and H-7/H-5a (*δ*_H_ 2.12) demonstrated the configurations of C-7 and C-8 as 7*S*∗ and 8S*, respectively. On the basis of the aforementioned observations and other detailed NOESY correlations (Figure 4), the structure of ehrenbergol A (**2**) was established.

The positive HRESIMS spectrum of ehrenbergol B (**3**) exhibited a pseudo molecular ion peak at *m/z* 387.2509 [M + Na]^+^, consistent with the molecular formula of C_22_H_36_O_4_, implying five degrees of unsaturation. The presence of two oxygenated methine [*δ*_H_ 3.40 (d, 1H, *J* = 7.6 Hz) and *δ*_C_ 85.1 (C-7); *δ*_H_ 3.32 (dd, 1H, *J* = 11.2, 2.0 Hz) and *δ*_C_ 80.1 (C-11)] implied that an ether linkage is present between C-7 and C-11, which was confirmed by the HMBC correlations from H-7 to C-11, and from H-11 to C-7. The NMR spectroscopic data (Table 3) indicated that **3** possesses a conjugated diene [*δ*_H_ 5.89 (1H, d, *J* = 5.0 Hz) and 5.99 (1H, d, *J* = 5.0 Hz); *δ*_C_ 150.2 (qC, C-1), 119.6 (CH, C-2), 122.3 (CH, C-3), and 137.1 (qC, C-4)] and an acetoxy [*δ*_C_ 169.2 (qC), 21.9 (CH_3_) and *δ*_H_ 1.67 (3H, s)];. The above functionalities account for three of the five degrees of unsaturation, implying that **3** is a cembranoid characterized by the presence of an ether linkage between C-7 and C-11.

**Table 3:** NMR data for compound **3**.

Position	*δ*_H_ *^a^* (*J* in Hz)	*δ*_C_ *^b^*, Type	HMBC	COSY	NOESY
1		150.2, qC			
2	5.89, br d (5.0)	119.6, CH	4, 14, 15	3	3, 15, 16, 18
3	5.99, br d (5.0)	122.3, CH	1, 5, 18	2, 18	2, 5a
4		137.1, qC			
5	2.16, m	39.6, CH_2_	3, 4, 6, 7	6a, 6b	3, 7, 6b, 18
6a	1.42, m	26.5, CH_2_	8	5a, 5b, 6b	5b, 19
6b	1.69, m		4, 5, 7	5a, 5b, 6a	
7	3.40, d (7.6)	85.1, CH	5, 6, 8, 9, 11, 19		5a, 9a
8		80.7, qC			
9a	1.73, m	35.4, CH_2_		9b, 10a, 10b	7, 9b, 11
9b	2.83, dt (12.4, 4.0)		19	9a, 10a, 10b	9a, 10b, 19
10a	1.56, m	23.0, CH_2_		10b	10b, 11
10b	1.39, m			10a	10b, 20
11	3.32, dd (11.2, 2.0)	80.1, CH	7, 12, 20	10a, 10b	9a, 10a, 14a
12		73.0, qC			
13a	1.46, m	41.0, CH_2_	20	13b, 14a	
13b	1.80, m		12, 20	13a	17, 20
14a	2.22, m	24.0, CH_2_	1, 2	13a, 13b, 14b	3, 11
14b	1.77, m		13	14a	
15	2.28, m	35.2, CH	16, 17	16, 17	2, 16, 17
16	1.08, d (7.2)	22.0, CH_3_	1, 15, 17	15	2, 15
17	1.10, d (6.4)	22.6, CH_3_	1, 15, 16	15	2, 13b, 15
18	1.64, s	17.2, CH_3_	3, 4, 5	3	2, 5b
19	1.50, s	17.1, CH_3_	7, 8, 9		6b, 9b
20	1.01, s	23.8, CH_3_	11, 12, 13		10b, 13b
OAc	1.67, s	169.2, qC			
		21.9, CH_3_			

*^a^* Spectra were measured in C_6_D_6_ (400 MHz); *^b^* Spectra were measured in C_6_D_6_ (100 MHz).

**Figure 6:**
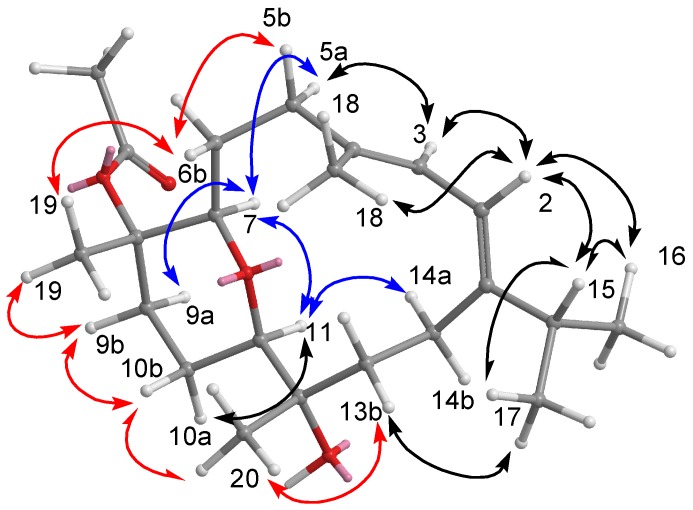
NOESY correlations of compound **3**.

The final assembly of **3** was determined by the information from COSY and HMBC experiments. The ^1^H-^13^C long-range correlations as determined from the HMBC spectrum allowed the connectivity of the structural fragments around each methyl group to be deduced (Figure 3). The crucial NOESY correlations (Figure 6) proved that the geometries of the conjugated diene at C-1/C-2 and C-3/C-4 were both *E*. The coupling constant (*J*_2,3_ = 5.0 Hz) further suggested the *s*-*cis* geometry of the above functionality [20,21]. The key NOESY correlations between H-3/H-14a (*δ*_H_ 2.22), H-14a/H-11, H-11/H-7, H-11/H-9a (*δ*_H_ 1.73), H-11/H-10a (*δ*_H_ 1.56), H-7/H-9b, H-7/H-3, H-7/H-5 (*δ*_H_ 2.16), Me-19/H-9b (*δ*_H_ 2.83), Me-20/H-10b (*δ*_H_ 1.39), and Me-20/H-13b (*δ*_H_ 1.80) suggested that H-7, and H-11 are on the same face (β), whereas Me-19 and Me-20 are oriented toward the other face (α), as shown in a computer generated 3D drawing. The above findings indicated the 7R*, 8S*, 11R*, and 12S* configurations as depicted in Figure 6. Therefore, the structure of **3** was elucidated as (7R*,8S*,11R*,12S*,1*Z*,3*E*)-8,12-dihydroxy-7,11-epoxycembra-1(2),3-diene 8-acetate. 

The cytotoxicities of metabolites **1**–**3** against P-388 (mouse lymphocytic leukemia), HT-29 (human colon adenocarcinoma) tumor cells, and human embryonic lung (HEL) cells are shown in Table 4. Metabolites **1**–**3** were also examined for antiviral activity against human cytomegalovirus (HCMV) using a human embryonic lung (HEL) and displayed antiviral activity against human cytomegalovirus, with IC_50s_ of 60, 46, and 5.0 μg/mL, respectively.

**Table 4:** Cytotoxicit and anti-HCMV activity of **1**–**3**.

Compounds	ED_50_ (μg/mL)				
	A549	HT-29	P-388	HEL	Anti-HCMV
1	20.8	>50	5.8	>50	60
2	>50	>50	7.4	>50	46
3	10.2	>50	4.7	>50	5.0

## 3. Experimental Section

### 3.1. General Experimental Procedures

Optical rotations were determined with a JASCO P1020 digital polarimeter. UV and IR spectra were obtained on JASCO V-650 and JASCO FT/IR-4100 spectrophotometers, respectively. NMR spectra were recorded on a Varian MR 400 NMR spectrometer at 400 MHz for ^1^H and 100 MHz for ^13^C. ^1^H NMR chemical shifts are expressed in *δ* (ppm) referring to the solvent peak *δ*_H_ 7.27 for CHCl_3_ or *δ*_H_ 7.15 for C_6_D_6_, and coupling constants are expressed in Hz. ^13^C NMR chemical shifts are expressed in *δ* (ppm) referring to the solvent peak *δ*_C_ 77.0 for CDCl_3_ or *δ*_C_ 128.0 for C_6_D_6_. MS were recorded by a Bruker APEX II mass spectrometer. Silica gel 60 (Merck, Germany, 230–400 mesh) and LiChroprep RP-18 (Merck, 40–63 μm) were used for column chromatography. Precoated silica gel plates (Merck, Kieselgel 60 F_254_, 0.25 mm) and precoated RP-18 F_254s_ plates (Merck) were used for thin-layer chromatography (TLC) analysis. High-performance liquid chromatography (HPLC) was carried out using a Hitachi L-7100 pump equipped with a Hitachi L-7400 UV detector at 220 nm together with a semi-preparative reversed-phased column (Merck, Hibar LiChrospher RP-18e, 5 μm, 250 × 25 mm).

### 3.2. Biological Material

The soft coral *S. ehrenbergi* was collected by SCUBA at San-Hsian-Tai, Taitong County, Taiwan, in July 2008 at a depth of 8 m and stored in a freezer until extraction. The voucher specimen (SST-13) was identified by Professor Chang-Feng Dai, National Taiwan University and deposited at the Department of Marine Biotechnology and Resources, National Sun Yat-sen University, Taiwan.

### 3.3. Extraction and Isolation

A specimen of soft coral *S. ehrenbergi* (4.0 kg) was minced and extracted with acetone (4 × 3 L) at room temperature. The combined acetone extracts were then partitioned between H_2_O and EtOAc. The resulting EtOAc extract (46.9 g) was subjected to gravity silica gel 60 column chromatography (Si 60 CC) using *n*-hexane and *n*-hexane/EtOAc of increasing polarity, to give 20 fractions. Fraction 15 (2.26 g), eluted with *n*-hexane/EtOAc (1:1), was further subjected to Si 60 CC (*n*-hexane/EtOAc, 7:1) to give 8 subfractions. A subfraction 15-4 (250 mg), was purified by RP-18 HPLC (MeOH/H_2_O, 75:25) to afford **2** (2.0 mg, 0.0005%).The fraction 14 (3.56 g), eluted with *n*-hexane/EtOAc (2:1), was further subjected to Si 60 CC (*n*-hexane/EtOAc, 8:1) to give 5 subfractions. A subfraction 14-2 (299 mg), was separated by a RP-18 flash column (MeOH/H_2_O, 60:40 to 100% MeOH) to give 6 fractions. The subfraction 14-2-6, eluted with MeOH/H_2_O (90:10), was purified by RP-18 HPLC (MeOH/H_2_O, 85:15) to afford **3** (2.4 mg, 0.0006%). A subfraction 14-3 (248 mg), was separated by a RP-18 flash column (MeOH/H_2_O, 50:50 to 100% MeOH) to give 7 fractions. The subfraction 14-3-3, eluted with MeOH/H_2_O (70:30), was purified by RP-18 HPLC (MeOH/H_2_O, 65:35) to afford **1** (3.2 mg, 0.0008%). 

(+)-12-Ethoxycarbonyl-11*Z*-sarcophine (**1**): White amorphous powder; 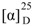 +77 (*c* 0.2, CHCl_3_); UV (MeOH) λ_max_ (log ε) 228 (3.72) nm; IR (neat) ν_max_ 3481, 2933, 1754, 1705, 1455, 1387, 1242, 1096, 991, 760 cm^−^^1^; ^1^H NMR (CDCl_3_, 400 MHz) and ^13^C NMR (CDCl_3_, 100 MHz) data in Table 1; HRESIMS *m/z* 397.1993 [M + Na]^+^ (calcd for C_22_H_30_O_5_Na, 397.1991).

Ehrenbergol A (**2**): White amorphous powder; 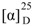 −184 (*c* 0.1, CHCl_3_); UV (MeOH) λ_max_ (log ε) 221 (3.72), 242 (3.32) nm; IR (neat) ν_max_ 3447, 2961, 2925, 2851, 1715, 1458, 1260, 1101, 1026, 799, 759 cm^−^^1^; ^1^H NMR (C_6_D_6_, 400 MHz) and ^13^C NMR (C_6_D_6_, 100 MHz) data in Table 1; HRESIMS *m/z* 371.2195 [M + Na]^+^ (calcd for C_21_H_32_O_4_Na, 371.2198).

Ehrenbergol B (**3**): White amorphous powder; 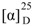 −84.0 (*c* 0.1, CHCl_3_); IR (neat) ν_max_ 3461, 2959, 1737, 1634, 1456, 1378, 1259, 1089, 1026, 801 cm^−^^1^; ^1^H NMR (C_6_D_6_, 400 MHz) and ^13^C NMR (C_6_D_6_, 100 MHz) data in Table 2; HRESIMS *m/z* 387.2509 [M + Na]^+^ (calcd for C_22_H_36_O_4_Na, 387.2511).

### 3.4. Cytotoxicity Assay

Cytotoxicity was determined on P-388 (mouse lymphocytic leukemia), HT-29 (human colon adenocarcinoma), and A-549 (human lung epithelial carcinoma) tumor cells using a modification of the MTT colorimetric method according to a previously described procedure [23,24,25]. The provision of the P-388 cell line was supported by J.M. Pezzuto, formerly of the Department of Medicinal Chemistry and Pharmacognosy, University of Illinois at Chicago. HT-29 and A-549 cell lines were purchased from the American Type Culture Collection. To measure the cytotoxic activities of tested compounds, five concentrations with three replications were performed on each cell line. Mithramycin was used as a positive control.

### 3.5. Anti-HCMV Assay

To determine the effects of natural products upon HCMV cytopathic effect (CPE), confluent human embryonic lung (HEL) cells grown in 24-well plates were incubated for 1 h in the presence or absence of various concentrations of tested natural products with three replications. Ganciclovir was used as a positive control. Then, cells were infected with HCMV at an input of 1000 pfu (plaque forming units) per well of a 24-well dish. Antiviral activity was expressed as IC_50_ (50% inhibitory concentration), or compound concentration required to reduce virus induced CPE by 50% after 7 days as compared with the untreated control. To monitor the cell growth upon treating with natural products, an MTT-colorimetric assay was employed [26,27].

## 4. Conclusion

The first investigation of soft coral *S. ehrenbergi* collected at San-Hsian-Tai (Taitong County, Taiwan) has led to the isolation of three new cembranoids, (+)-12-ethoxycarbonyl-11*Z*-sarcophine (**1**) as well as ehrenbergol A and B (**2** and **3**). Metabolites **1**–**3** were not cytotoxic towards P-388 (mouse lymphocytic leukemia), HT-29 (human colon adenocarcinoma) tumor cells, and human embryonic lung (HEL) cells. However, metabolites **1**–**3** displayed antiviral activity towards human cytomegalovirus, with IC_50_s of 60, 46, and 5.0 μg/mL, respectively. Ehrenbergol B is the first cembranoid from Taiwanese soft corals to show potent anti-HCMV activity.

## Supplementary Files

Supplementary File 1: PDF-Document (PDF, 245 KB)
