# Supplementary material for: Three New Cembranoids from the Taiwanese Soft Coral Sarcophyton ehrenbergi
Source: Mar Drugs. 2012 Jun 27;10(7):1433–44. doi: 10.3390/md10071433 (PMC3407922; doi:10.3390/md10071433)

Supplementary Information

Table of Contents

Figure S1. ^{1}H NMR spectrum (400 MHz) of (+)-12-ethoxycarbonyl-11*Z*-sarcophine (**1**) in $CDCl_3$. 2

Figure S2. ^{13}C NMR spectrum (100 MHz) of (+)-12-ethoxycarbonyl-11*Z*-sarcophine (**1**) in $CDCl_3$. 3

Figure S3. ^{1}H NMR spectrum (400 MHz) of ehrenbergol A (**2**) in C_6D_6. 4

Figure S4. ^{13}C NMR spectrum (100 MHz) of ehrenbergol A (**2**) in C_6D_6. 5

Figure S5. ^{1}H NMR spectrum (400 MHz) of ehrenbergol B (**3**) in C_6D_6. 6

Figure S6. ^{13}C NMR spectrum (100 MHz) of ehrenbergol B (**3**) in C_6D_6. 7

Figure S1. ^{1}H NMR spectrum (400 MHz) of (+)-12-ethoxycarbonyl-11*Z*-sarcophine (**1**) in $CDCl_3$.

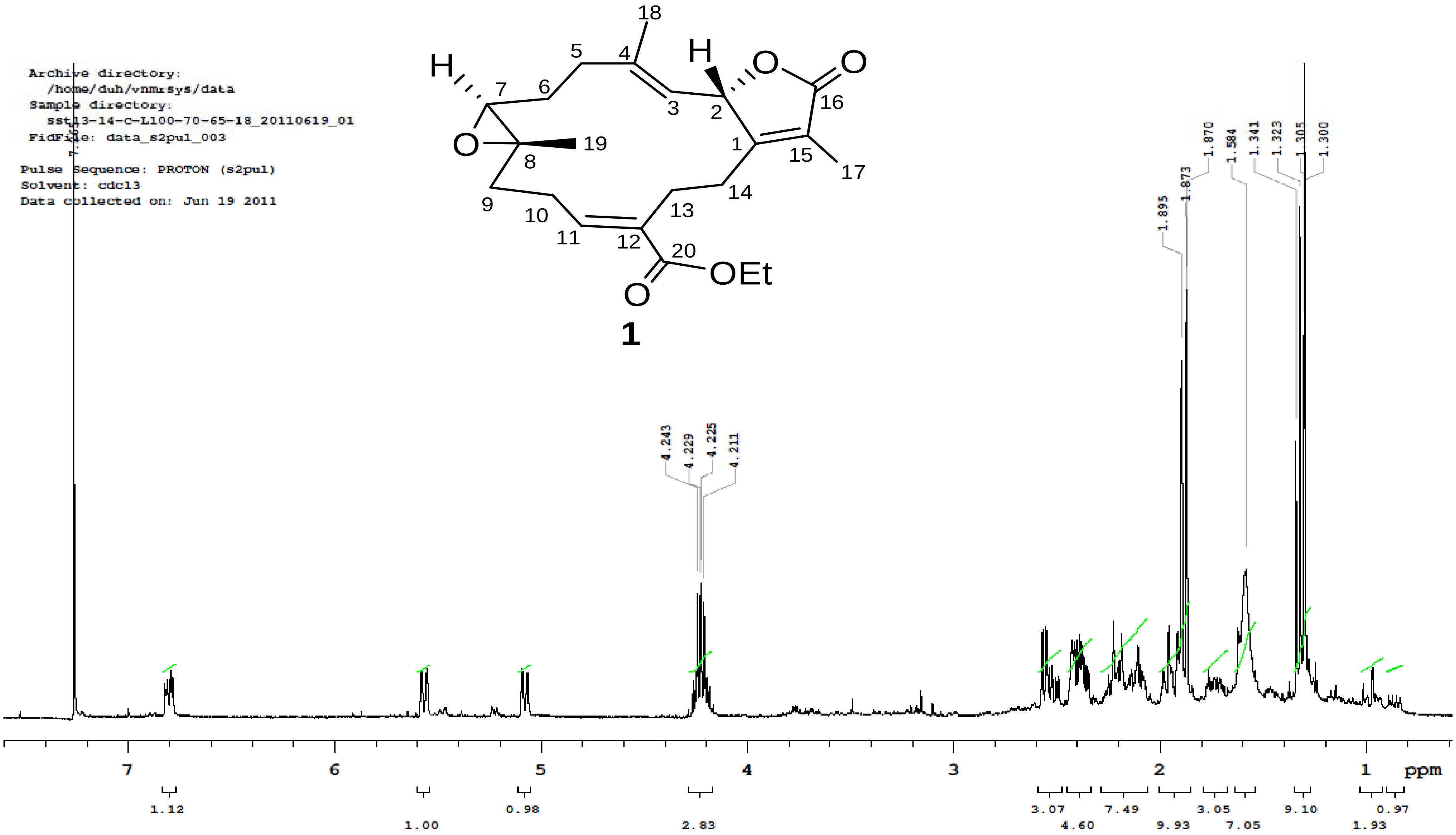

Figure S2. ^{13}C NMR spectrum (100 MHz) of (+)-12-ethoxycarbonyl-11*Z*-sarcophine (**1**) in $CDCl_3$.

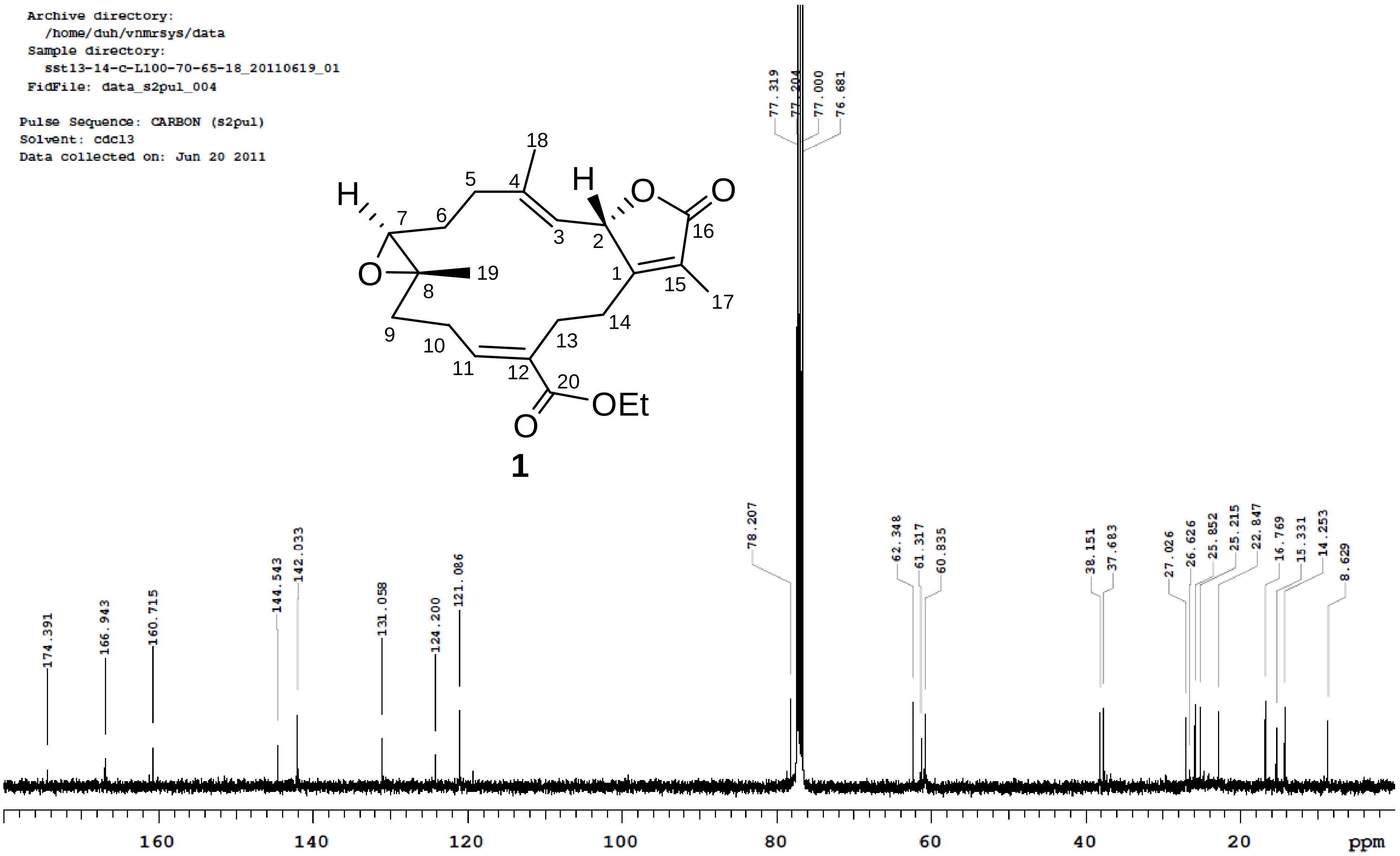

Figure S3. ^{1}H NMR spectrum (400 MHz) of ehrenbergol A (**2**) in C_6D_6.

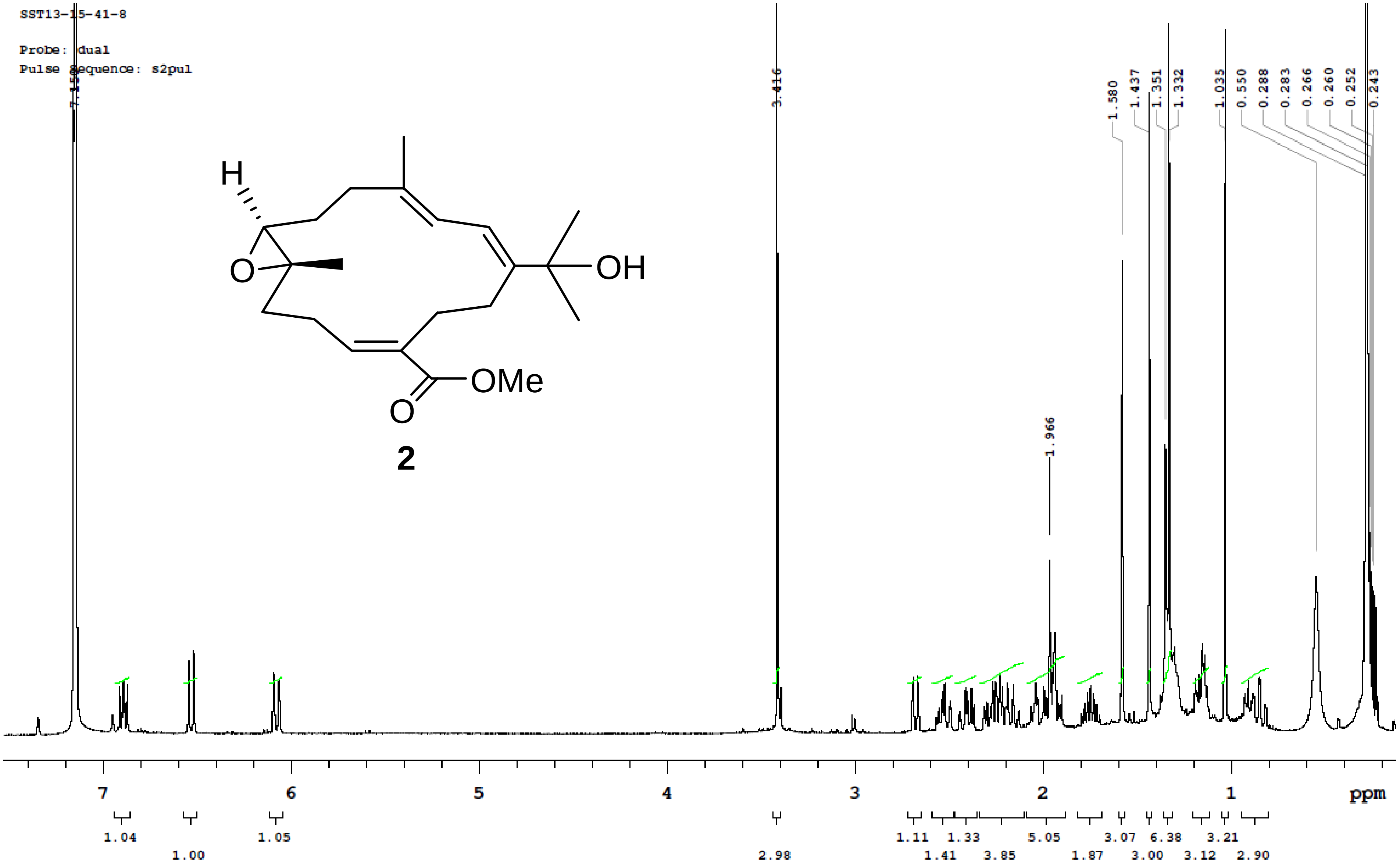

Figure S4. ^{13}C NMR spectrum (100 MHz) of ehrenbergol A (**2**) in C_6D_6.

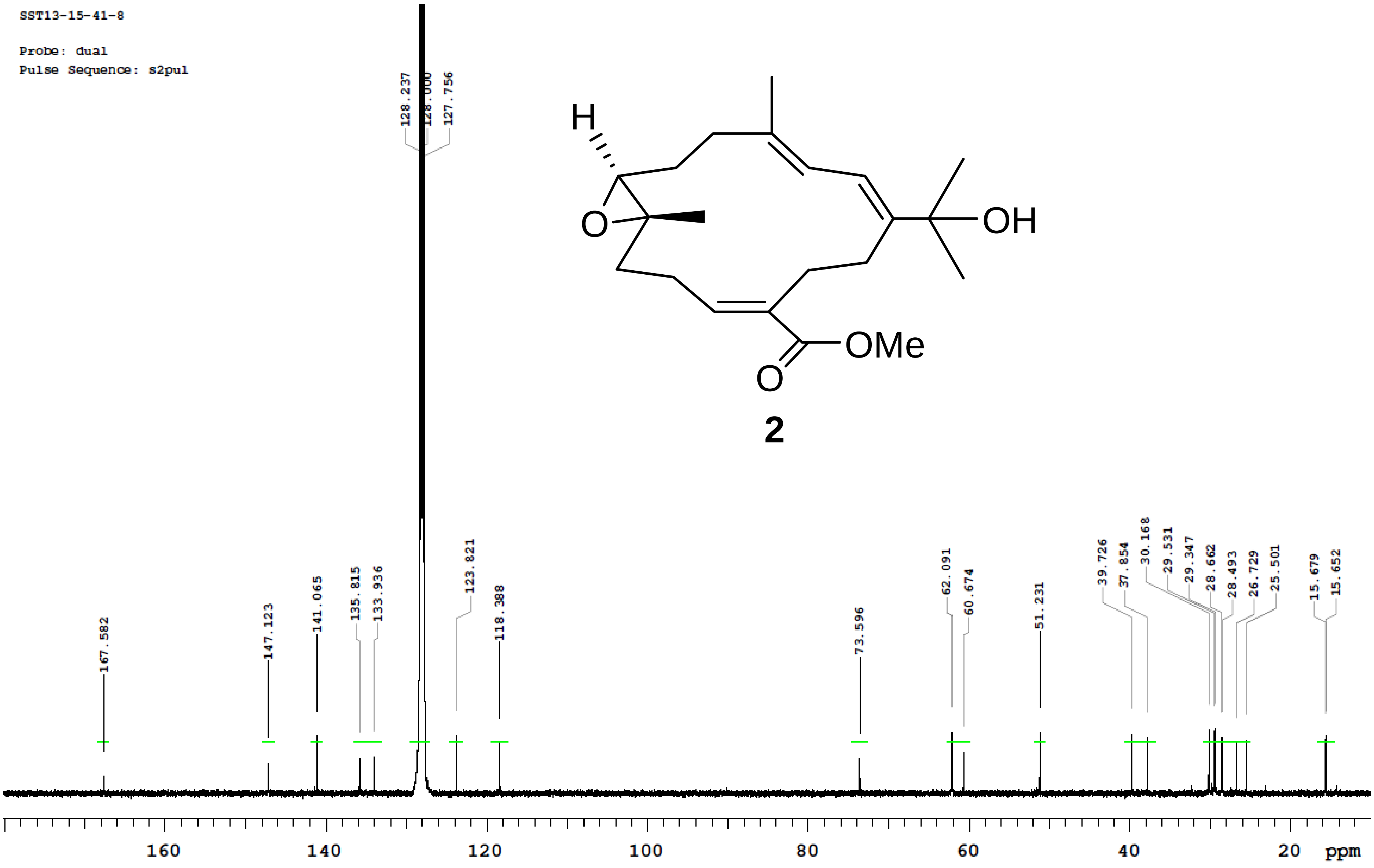

Figure S5. ^{1}H NMR spectrum (400 MHz) ehrenbergol B (**3**) in C_6D_6.

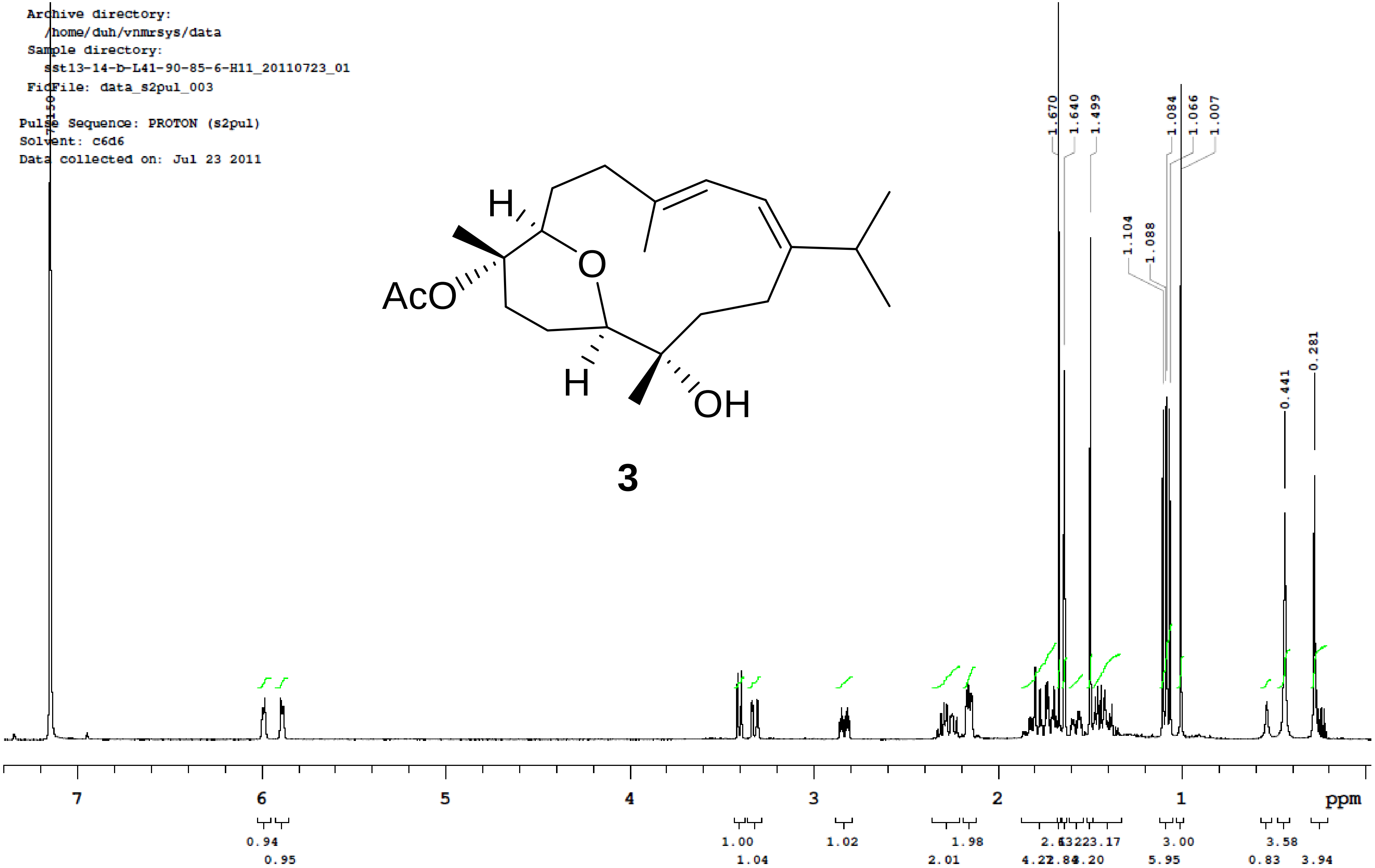

Figure S6. ^{13}C NMR spectrum (100 MHz) ehrenbergol B (**3**) in C_6D_6.

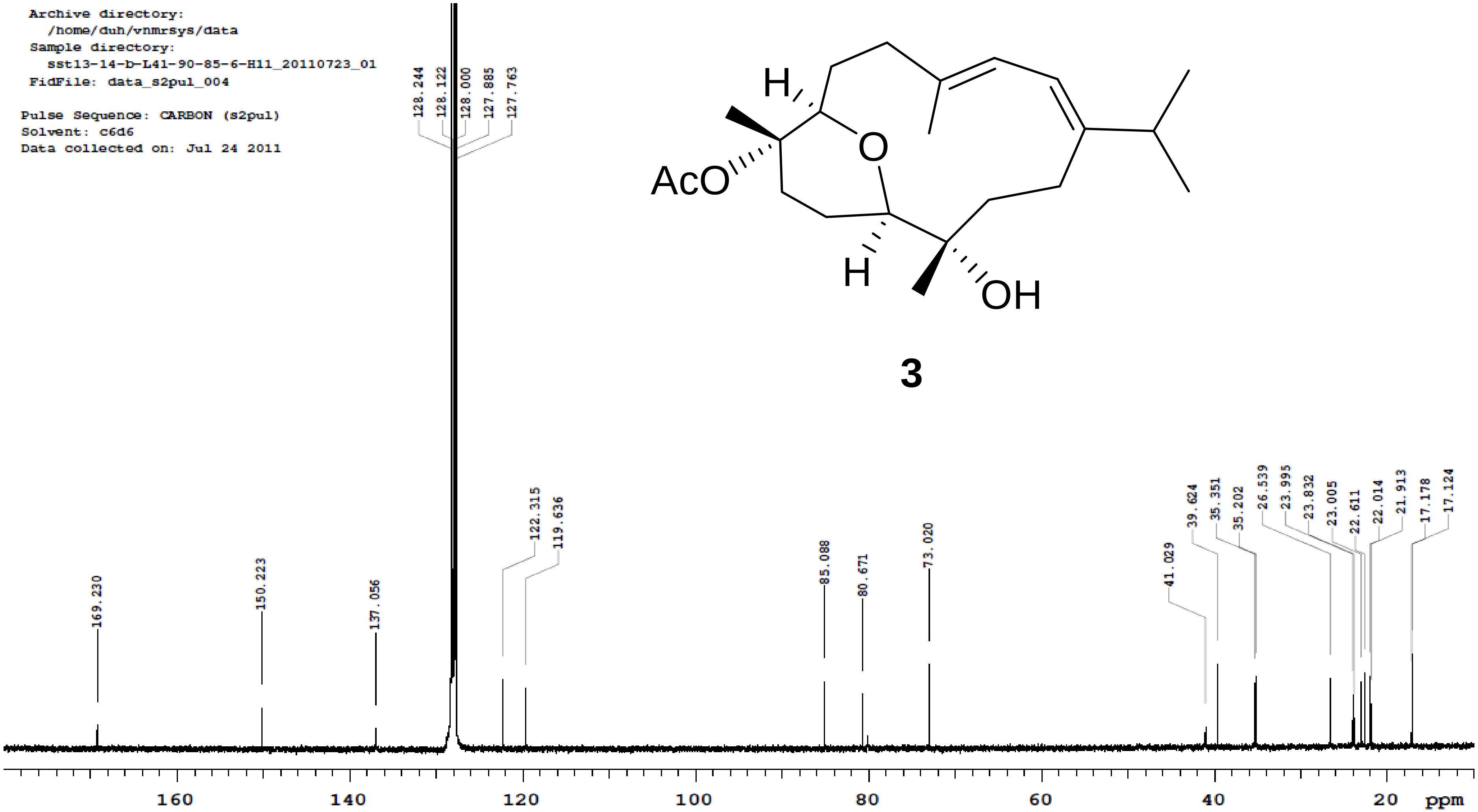

Supplement: Supplementary File 1: — PDF-Document (PDF, 245 KB) [file marinedrugs-10-01433-s001.pdf]
